# Combination of gemcitabine-containing magnetoliposome and oxaliplatin-containing magnetoliposome in breast cancer treatment: A possible mechanism with potential for clinical application

**DOI:** 10.18632/oncotarget.9671

**Published:** 2016-05-27

**Authors:** Hui Ye, Jiansong Tong, Jiangyi Liu, Wenman Lin, Chengshou Zhang, Kai Chen, Jie Zhao, Wenjing Zhu

**Affiliations:** ^1^ School of Basic Medical Sciences, Wenzhou Medical University, Wenzhou, Zhejiang, 325035, China; ^2^ Department of Cellular and Molecular Biology, The Scripps Research Institute, La Jolla, CA 92037, USA; ^3^ School of Ophthalmology and Optometry, Wenzhou Medical University, Wenzhou, Zhejiang, 325035, China; ^4^ School of Renji, Wenzhou Medical University, Wenzhou, Zhejiang, 325035, China; ^5^ School of the First Clinical Medical Sciences, Wenzhou Medical University, Wenzhou, Zhejiang, 325035, China

**Keywords:** gemcitabine, oxaliplatin, magnetoliposome, breast cancer, MCF-7

## Abstract

Breast cancer is a major global health problem with high incidence and case fatality rates. The use of magnetoliposomes has been suggested as an effective therapeutic approach because of their good specificity for cancers. In this study, we developed two novel magnetoliposomes, namely, Gemcitabine-containing magnetoliposome (GML) and Oxaliplatin-containing magnetoliposome (OML). These magnetoliposomes were combined (CGOML) was used to treat breast cancer under an external magnetic field. Biosafety test results showed that GML and OML were biologically safe to blood cells and did not adversely affect the behavior of mice. Pharmacokinetic and tissue distribution studies indicated that both magnetoliposomes exhibited stable structures and persisted at the target area under an external magnetic field. Cell and animal experiments revealed that CGOML can markedly suppress the growth of MCF-7 cells, and only the CGOML group can minimize the tumor size among all the groups. Finally, CGOML can significantly inhibit MCF-7cell growth both in vitro and vivo by activating the apoptotic signaling pathway of MCF-7 cells.

## INTRODUCTION

Breast cancer is the most frequently diagnosed malignancy and a major cause of cancer-related deaths among women. This malignancy accounts for 25% of total cancer cases and 15% of cancer deaths worldwide [1–3]. Hence, the prevention of breast cancer has been extensively investigated. However, the morbidity of breast cancer has been increasing in most countries and projected to further rise over the next 20 years [4–7]. Breast cancer is usually treated by surgery, chemotherapy, radiotherapy, and endocrinotherapy. Among these therapies, chemotherapy plays an important role in the four stages of breast cancer, namely, preoperative neoadjuvant therapy, postoperative adjuvant therapy, recurrence, and metastasis.

**Figure 1:**
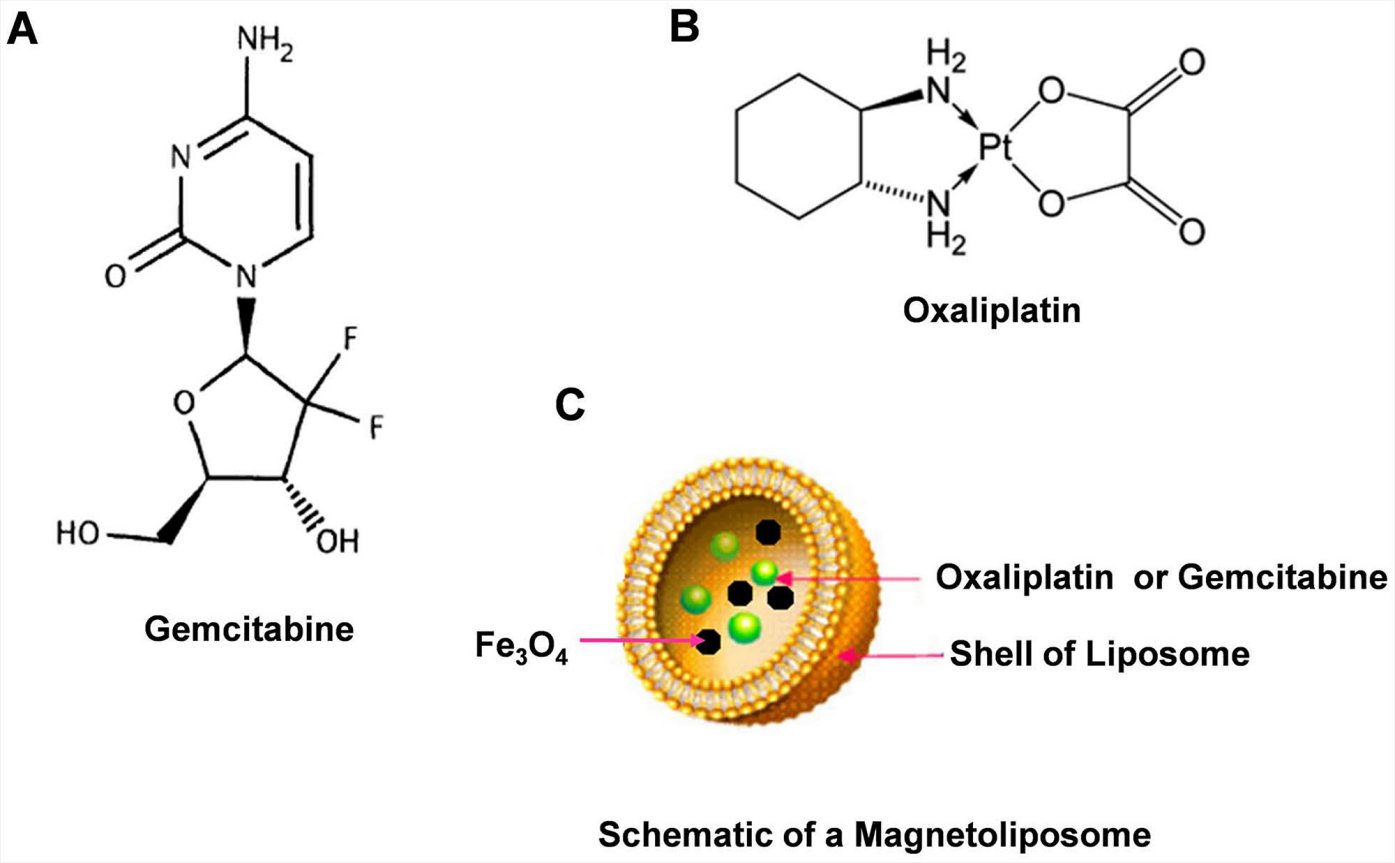
Chemical structures of Gemcitabine, Oxaliplatin and schematic of magnetoliposome Notes: **A.** Chemical structures of Gemcitabine. **B.** Chemical structures of Oxaliplatin. **C.** schematic of magnetoliposome.

The combined therapy of gemcitabine (Figure 1A) and oxaliplatin (Figure 1B) (GemOx) has exhibited satisfactory efficacy, preventing recurrence. This therapy has become an effective solution against drug resistance in tumors. Gemcitabine and platinum derivatives were reported to demonstrate obvious synergy in breast cancer treatment [8, 9]. Gemcitabine suppresses DNA repair mechanisms involved in biological resistance to platinum agents. However, previously used cytotoxic compounds do not contain standard adjuvants or metastatic breast cancer (MBC) regimens. Thus, these agents are expected to lack cross resistance in clinical settings. These two drugs are expected to yield the lowest overlapping toxicities. The clinical application of combined gemcitabine and cisplatin [8, 10] or carboplatin [11, 12], similar to the combination of oxaliplatin and gemcitabine in apancreatic cancer model [13, 14], is apparently feasible and a desirable solution to the aforementioned concerns.

Nonetheless, several studies have indicated that the side effects of gemcitabine and oxaliplatin restrict their clinical curative effect. These two drugs can cause bone marrow suppression, gastrointestinal tract reaction, alopecia, influenza-like symptoms, neurotoxicity, edema, allergic reactions, renal toxicity [15], neurotoxicity, cardiotoxicity, gastrointestinal reactions, hemorrhage, and hypersensitivity [16, 17]. Thus, the use of targeted drugs combined with various therapeutic agents has been recommended as a favorable therapeutic strategy [18].

The curative effect of traditional targeted therapy on solid tumors is restricted by intravenous emulsions, nanoparticles, and common liposomes [19–21]. Thus, magnetic nanoparticles have attracted considerable interest in targeted therapy for cancers [22–25]. Among these particles, magnetic liposomes (also called magnetoliposomes, MLs) have been considered to represent a novel drug delivery system for cancer drug targeting [26]. Liposomes can be gradually biodegraded in the body because of their good half-life. Furthermore, drugs in magnetic liposomes can be guided under an external magnetic field to a target area and slowly released onsite [22, 26]. Compared with systemic chemotherapy, ML therapy can obviously enhance the efficacy of drugs and reduce their side effects.

In this work, we conducted a pilot research on a novel magnetoliposome containing gemcitabine (or oxaliplatin) (Figure 1C). We further evaluated the effects of CGOML on the growth inhibition of MCF-7 cells (i.e., cell level) and tumors in nude mice bearing breast cancer (MCF-7) (i.e., animal level). Finally, we proposed a possible model of targeted therapy and signaling pathway involved in CGOML-induced apoptosis of MCF-7 cells.

## RESULTS

### Preparation and characterization of GML and OML

**Figure 2:**
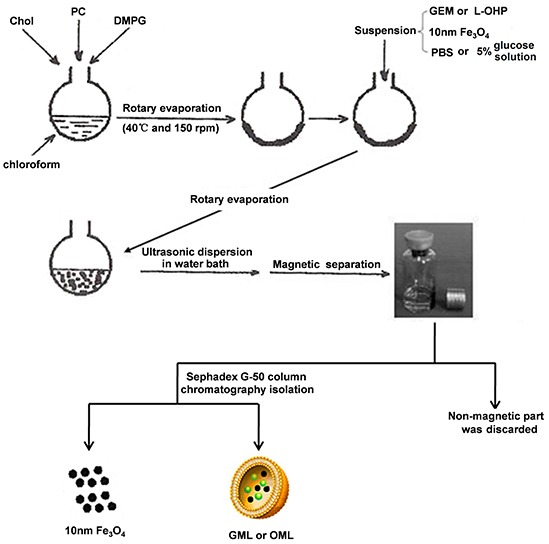
Explains the steps involved in preparation of GML, OML by reverse-phase evaporation followed by water bath ultrasonication Note: Chol, PC, DMPG, GEM, L-OHP, PBS, GML and OML are Cholesterol, Egg yolk phosphatidylcholine, Dimyristoyl phosphatidyl glycerol, Gemcitabine, Oxaliplatin, Phosphate Buffer Solution, Magnetoliposome containing gemcitabine and Magnetoliposome containing oxaliplatin, respectively.

GML or OML was prepared by reverse-phase evaporation, followed by water-bath ultrasonication (Figure 2). After magnetic sorting, GML or OML was further separated from free gemcitabine (or oxaliplatin) by Sephadex G-50 mini-columns. Subsequently, we tested the mean diameter, PI, encapsulation efficiency, and in vitro release of the GML or OML samples with three lipid compositions, namely, phosphatidylcholine (PC), cholesterol (Chol), and dimyristoyl phosphatidyl glycerol (DMPG) (PC/Chol/DMPG: 6:3:0, 6:4:1, and 6:5:1). The results showed that the optimal molar ratio of PC/Chol/DMPG was 6:4:1. The mean diameter of GML or OML was approximately 227.6 or 169.3 nm. The PI of GML or OML was close to 0.133 or 0.0597, and their drug-encapsulation efficiency was 73.5% or 78.2%. By contrast, the encapsulation efficiency of ferroferric oxide in MLs without any drug was 68.3%. Supplementary Figure S1 shows the in vitro release profiles of OML and GML at 37°C in 5% glucose solution. The releasing dosage rate of the OML group (58%) in the first 2 h was significantly lower than that of the free oxaliplatin group (95%) (p<0.05). Similarly, the releasing dosage rate in the GML group (45.5%) was significantly lower than that of the free gemcitabine group (90%) (p<0.05). Subsequently, 42% of the drug can be well-sustained-released from OML after 2 h (i.e., after a sudden release in initial phase). Similarly, 54.5% of the drug can be well-sustained-released from GML.

**Figure 3:**
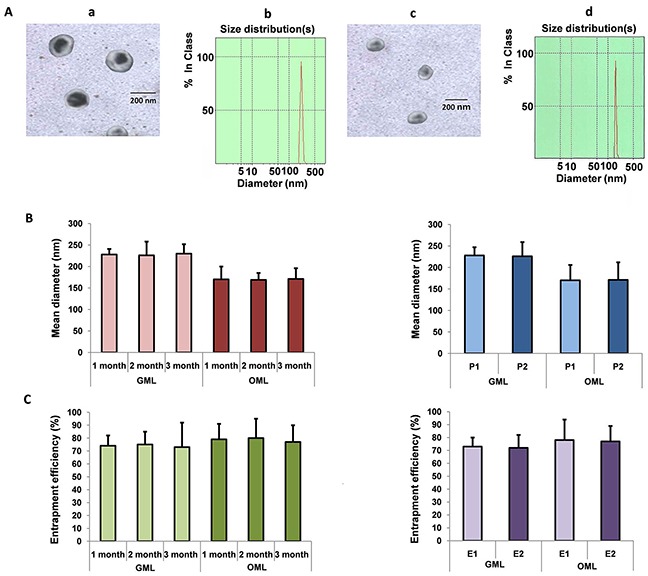
Preparation and characterization of GML and OML Notes: **A.** Transmission electron microscopy image and diameter distribution of GML and OML:Bar=200 nm, the mean diameter of GML, OML was 227.6 nm, 169.3 nm, respectively (GML (a panel), OML (c panel)); Diameter distribution of GML, OML particles were determined using dynamic light scattering method, respectively (GML (b panel), OML (d panel)). **B.** The mean diameter of the GML, OML particle after storage and irradiation sterilization: The mean diameter of GML, OML did not show significant difference when GML, OML was lyophilized with 5% mannitol as protective agent and stored at room temperature within three months (left panel); The mean diameter of the GML, OML particle did not also show significant difference before and after irradiation sterilization. P1 represents the GML, OML particle before irradiation sterilization, while P2 is the GML, OML particle after irradiation sterilization (right panel). **C.** Entrapment efficiency of the GML, OML particle after storage and irradiation sterilization: The drug entrapment efficiency did not change significantly when GML, OML was lyophilized with 5% mannitol as protective agent and stored at room temperature within three months (left panel); Effect of irradiation sterilization on drug entrapment efficiency of GML, OML did not also change significantly. E1 is the entrapment efficiency before irradiation sterilization while E2 represents the entrapment efficiency after irradiation sterilization (right panel).

DLS results showed that the GML and OML particles were almost spherical and smooth, with mean diameters of approximately 227.6 nm [Figure 3A (a and b panels)] and 169.3 nm [Figure 3A (c and d panels)], respectively. Afterward, a Nd_2_Fe_12_B magnet was placed near the ampoule, and almost all GML or OML particles moved toward the side close to the magnet within 30 s (GML) or 32 s (OML). However, the GML and OML solutions were homogeneous without the Nd_2_Fe_12_B magnet near the ampoule. Up to 2 mL of the sample was lyophilized with 5% mannitol as a protective agent and stored at room temperature for 1–3 months, during which no significant differences were found among the GML or OML groups (left panels in Figures 3B and 3C). Similarly, the particle sizes and entrapment efficiencies of GML and OML neither increased nor decreased by irradiation sterilization (right panels in Figures 3B and 3C).

### Biological safety of GML and OML to cells and animals

All hemolysis rates in tubes containing GML or OML were lower than 5% (Tables 1 and 2). Supplementary Figure S2 and Tables S1 and S2 show no significant differences in the platelet aggregation rate, change in the absolute numbers of leukocytes, and phagocytic activity of leukocytes between the control and experimental groups. Table 3 also shows that all mice in the GML groups showed active behavior, such as catching ear, licking feet, and stirring head. Compared with mice in the saline group, mice in the GML groups demonstrated good appetite and normal excrement grain, as well as exhibited shiny fur and fleshy red claws and tails (Table 3). All mice normally behaved after administration of different doses of GML, with 0% mortality. The biological safety of OML to animals was also determined using the aforementioned method, and the results are also listed in Table 3. The resultsof animal acute toxicity from GML and OML were exactly the same.

**Table 1:** GML hemolysis test results (n=5)

Group	ODt-ODnc	ODpc-ODnc	Hemolysis rate (%)	Macroscopic observation
1	0.0154	0.456	3.38	−
2	0.0166	0.456	3.64	−
3	0.0170	0.456	3.73	−
4	0.0181	0.456	3.97	−
5	0.0192	0.456	4.21	−
6	0.0196	0.456	4.30	−

Note: –, no hemolysis.

**Table 2:** OML hemolysis test results (n=5)

Group	ODt-ODnc	ODpc-ODnc	Hemolysis rate (%)	Macroscopic observation
1	0.0143	0.441	3.24	−
2	0.0155	0.441	3.51	−
3	0.0162	0.441	3.67	−
4	0.0169	0.441	3.83	−
5	0.0180	0.441	4.08	−
6	0.0187	0.441	4.24	−

Note: –, no hemolysis.

**Table 3:** The observation item and possible results of animal acute toxicity

Observation item	Possible results by observation
Spontaneous activity	Increase or decrease, lying motionless for reducing tiredness or bouncing up and down
Muscle tension	Increase or decrease, myotonia or muscle relaxation
Muscular movement	tremor, convulsion, paralysis, ataxia
Reaction	lagsinresponse, nervousness
Breathing	tachypnea, suppression, respiratory failure
Autonomic nerve movement	lachrymation, bristling, exorbitism, salivation, diarrhea, writhing reaction
Skin color	cyanosis, pallor, hyperaemia
Death time	slow dying, sudden death
Death symptoms	opisthotonos, struggle, froth at the mouth

### Pharmacokinetic and tissue distribution studies

**Figure 4:**
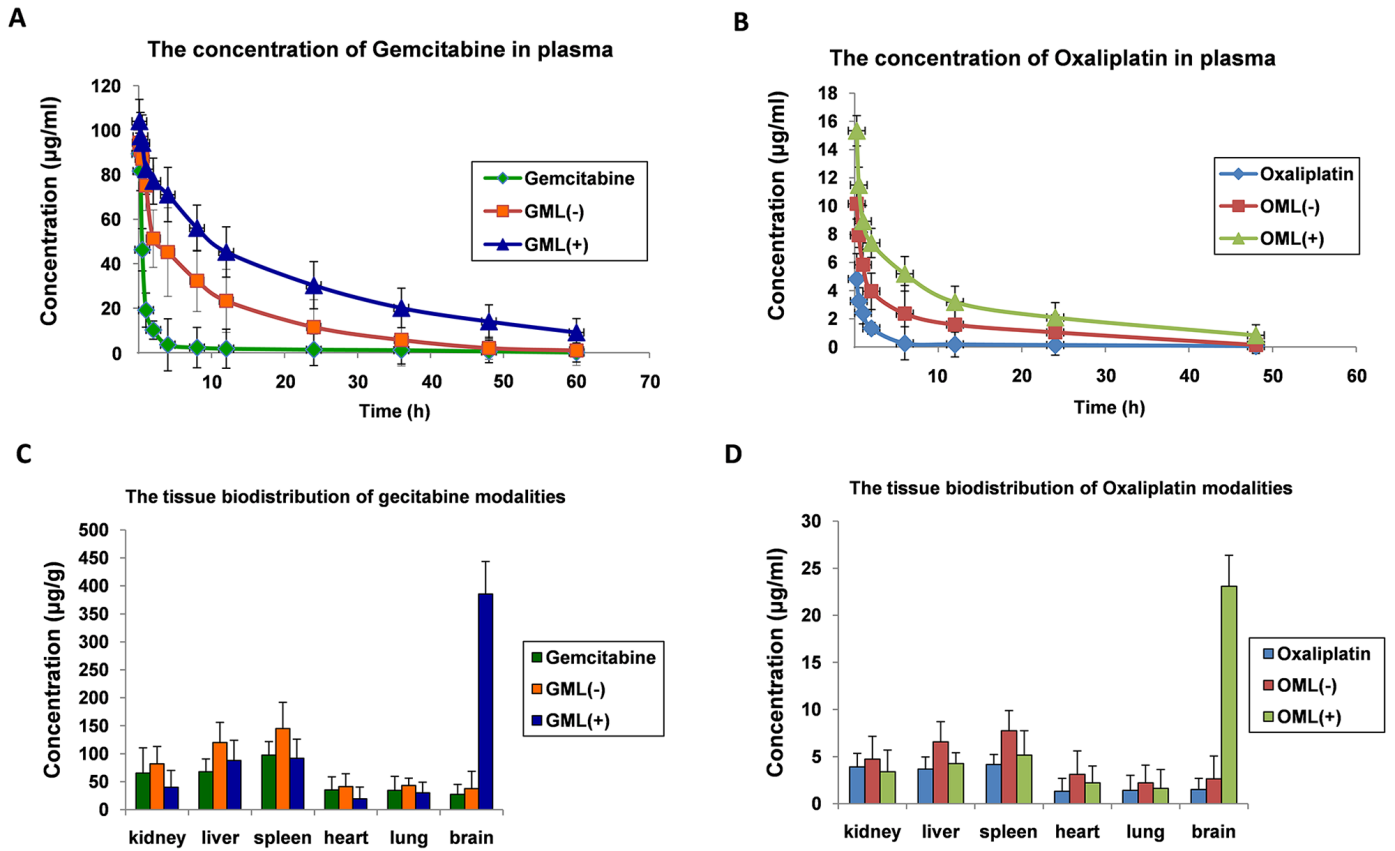
Pharmacokinetic and tissue-distribution studies of GML, OML in mice **A.** plot of gemcitabine concentrations in plasma at various time points from three groups of mice intravenously injected with free gemcitabine, GML (+)(namely: the heads of the mice were placed into a continued external magnetic field of 5000 GS for 30 min after mice intravenously injected with GML), and GML (−) (namely: the heads of the mice were not placed into a continued external magnetic field after mice intravenously injected with GML), respectively. The dosage of gemcitabine was 35 mg/kg in all experimental mice. The gemcitabine concentrations were measured by HPLC. The values were expressed as mean ± SD (n=10). **B.** In the same way, pharmacokinetic studies of OML in mice were determined by above same methods, and the dosage of oxaliplatin was 5 mg/kg in all experimental mice. **C.** three groups of mice intravenously injected with free gemcitabine, GML(+), and GML(−). For GML (+) group, the heads of the mice were placed into a continued external magnetic field of 5000 GS for 30 min. 90 min after the injection, the gemcitabine concentrations in the different tissues including the kidneys, liver, spleen, heart, lungs and brain were measured by HPLC. The values were expressed as mean ± SD (n=10). Compared with other tissue, *P<0.05. **D.** In the same way, tissue-distribution studies of OML in mice were determined by above same methods.

Figure 4A shows that the plasma gemcitabine levels in all the groups continuously increased and reached the maximum concentrations after 30 min but gradually decreased with time. The concentrations of gemcitabine in liposomes modalities were much higher than those of free gemcitabine at various time points in the plasma (p<0.05). Additionally, GML (+) presented higher concentration of gemcitabine in plasma than that shown by GML (−). This finding indicated that GML under magnetic field decreased much slower than that without a magnetic field (p < 0.05). These results suggested that a magnetic field prolongs the circulation of GML because of the avoidance of uptake by the reticuloendothelial system (RES). In addition, the results of pharmacokinetic studies on OML were consistent with those on GML (Figures 4A and 4B).

Figure 4C illustrates the tissue biodistribution of gemcitabine, in which the concentration in the brain is shown to be much higher than those in the kidneys, liver, spleen, heart, or lungs. Similarly, the results of tissue distribution studies on OML were consistent with those on GML (Figures 4C and 4D). Therefore, these results suggested that magnetic materials can increase the specific affinity of drugs. GML and OML can accumulate in a targeted tissue through an external magnetic field.

### Cellular uptake of oxaliplatin and detection of platinum-DNA adducts

**Figure 5:**
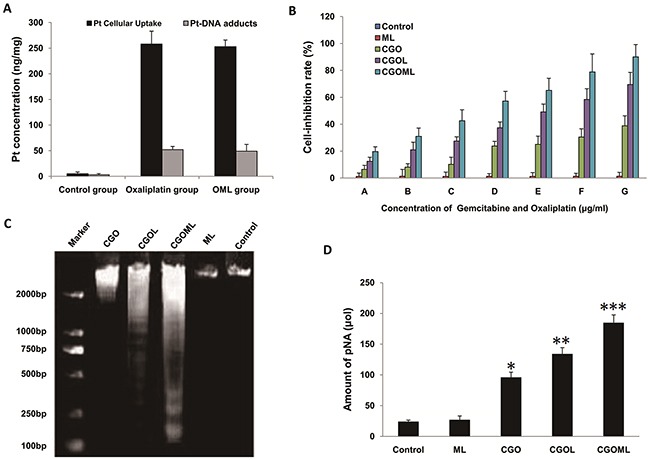
Cellular uptake of oxaliplatin, detection of platinum-DNA adducts and CGOML inhibit the growth of Breast cancer MCF-7 cells Notes: **A.** Comparison of in vitro cellular platinum uptake (solid black bars) and platinum-DNA adducts (open bars) among cells of control group (0 μg oxaliplatin/ml), Oxaliplatin group (20 μg oxaliplatin/ml) and OML group (20 μg oxaliplatin/ml) for 6 h at 37°C. The intracellular concentration of platinum in oxaliplatin group and OML group were much higher than those of control group *(p < 0.01)* and the number of platinum-DNA adducts in oxaliplatin group and OML group were much higher than those of control group *(p < 0.01)*. **B.** Inhibitory rates of MCF-7 cells in the experimental (ML, CGO, CGOL, and CGOML) groups and control group. Note: A, B, C, D, E, F and G respectively represent different concentration combinations of gemcitabine and oxaliplatin in the experimental groups (i.e., A=0.5μg gemcitabine/ml + 0.5 μg oxaliplatin/ml, B=1μg gemcitabine/ml + 1μg oxaliplatin/ml, C=2μg gemcitabine/ml + 1.5μg oxaliplatin/ml, D=4μg gemcitabine/ml + 2.5μg oxaliplatin/ml, E=8μg gemcitabine/ml + 5μg oxaliplatin/ml, F=16μg gemcitabine/ml + 10μg oxaliplatin/ml, G=32μg gemcitabine/ml + 20μg oxaliplatin/ml), but control group: 0 μg gemcitabine/ml + 0 μg oxaliplatin/ml. **C.** DNA ladder assay results of MCF-7 cells treated with Control, ML, CGO, CGOL, and CGOML for 24 hours. **D.** concentrations of pNA from 5 different groups treated with Control, ML, CGO, CGOL, and CGOML were examined by determining optical density values at 405 nm with colorimetric kits, *p < 0.05, **p < 0.02, ***p < 0.01, significantly different from control group.

Oxaliplatin forms inter-strand and intra-strand platinum-DNA adducts that induce several signal transduction pathways leading to apoptosis [27]. In this study, the intracellular concentrations of platinum in the control, oxaliplatin, and OML groups were 5, 258, and 253 ng/mg protein, respectively (Figure 5A). Thus, the intracellular concentrations of platinum in the oxaliplatin and OML groups were much higher than those in the control group (p<0.01). However, no significant difference was found between the oxaliplatin and OML groups (p>0.05). The control, oxaliplatin, and OML groups presented 3, 52, and 49 ng/mg platinum-DNA adducts, respectively. Thus, more platinum-DNA adducts were found in the oxaliplatin and OML groups than in the control group (p<0.01). However, no significant difference was found between the oxaliplatin and OML groups (p>0.05). These results indicated that OML also efficiently formed inter-strand and intra-strand platinum-DNA adducts (Pt-GG and Pt-AG) as free oxaliplatin.

### CGOML inhibits cell growth

Compared with the control group, the CGO, CGOL, and CGOML groups inhibited the MCF-7 cell growth in adose-dependent manner, but ML treatment only slightly inhibited the cell growth (Figure 5B). The inhibitory rates of the CGOL and CGOML groups were obviously higher than that of the CGO group, with significant difference between the CGOL and CGOML groups. These results demonstrated that encapsulation of gemcitabine or oxaliplatin with hydrophobic liposome can promote the delivery of gemcitabine or oxaliplatin into living cells. Significantly, these results proved that the drug encapsulated in ML can more efficiently suppress tumor cell growth than the free drug.

Gel electrophoretic analysis of internucleosomal DNA fragmentation demonstrated the presence of primarily high-molecular-weight DNA, as indicated by the absence of drug treatment (control and ML groups). However, a DNA ladder pattern, which is typical of apoptosis, was distinctly observed in the CGO, CGOL, and CGOML groups during drug-induced cell apoptosis. Figure 5C shows that the CGOML group exerted the strongest drug effect on living cells.

Caspase-3 activity in cells during apoptosis can be analyzed using a caspase-3 colorimetric kit. The CGOML group, among all the groups, obtained the highest pNA amount, which was approximately 7.7 times that of the control group. By contrast, the pNA amounts in the CGO and CGOL groups were approximately 4 and 5.6 times that of the control group, respectively (Figure 3D). No significant difference was found in the pNA amount between the control and ML groups. These results further demonstrated that CGOML exerted the greatest effect on cell-growth inhibition.

### CGOML inhibits the growth of breast tumors

**Figure 6:**
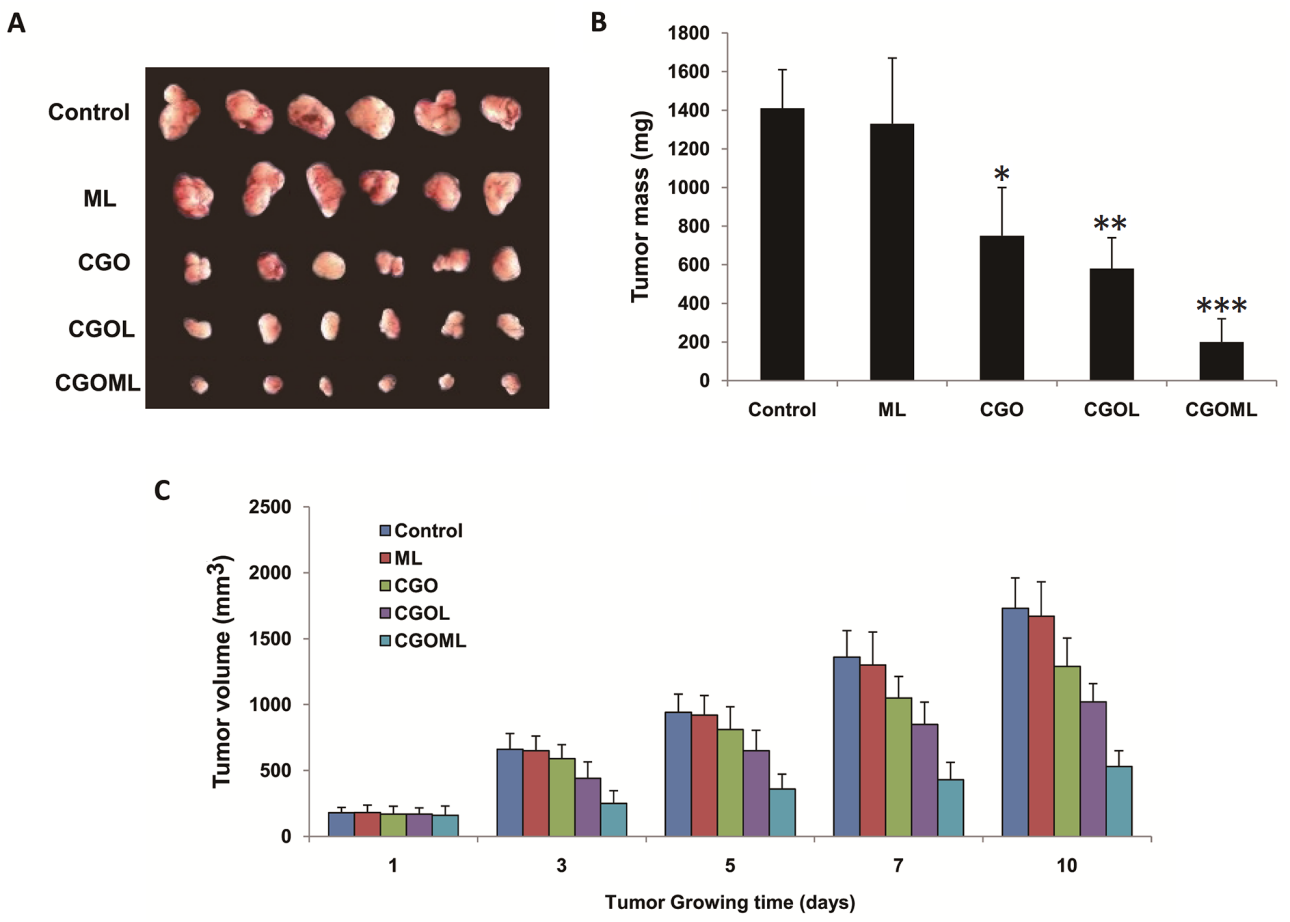
CGOML inhibits breast tumor growth Notes: **A.** Tumors in different groups was completely collected from nude mice. **B.** Comparison of tumor masses from nude mice among different groups, *p < 0.05, **p < 0.02, ***p < 0.01, significantly different from control group(n=6). **C.** Plot of tumor volumes from nude mice among different groups (n=6). Note: the drug concentration of each group respectively was Control (0 μg Gemcitabine/g, 0 μg Oxaliplatin/g), ML (0 μg Gemcitabine/g, 0 μg Oxaliplatin/g), CGO (35 μg Gemcitabine/g, 5 μg Oxaliplatin/g), CGOL (35 μg Gemcitabine/g, 5 μg Oxaliplatin/g), and CGOML (35 μg Gemcitabine/g, 5 μg Oxaliplatin/g).

Tumor sizes were obviously reduced in the CGOL, CGO, and CGOML groups, whereas no change was found in the ML group compared with the control group (Figure 6A). Tumor sizes were determined at 1, 3, 5, 7, and 10 days after treatment (Figures 6B and 6C). Tumor sizes obviously decreased in all groups but not in the control and ML groups. The largest reduction was observed in the CGOML group, with mean weight of tumors at approximately 201 mg, which was markedly lower than those in the CGO (750 mg) and CGOL (582 mg) groups. CGOML significantly reduced tumor sizes (p<0.01), which was 85.8% of the control group. Figure 6 shows no significant difference in tumor inhibition between the ML and control groups. The mean tumor weight in the ML group was approximately 1335 mg, which was almost equal to the mean tumor weight in the control group (1410 mg). Thus, our data proved that targeted treatment using CGOML can greatly enhance drug effects to treat MCF-7 (Figure 6).

### CGOML activates cell apoptosis in breast tumors

**Figure 7:**
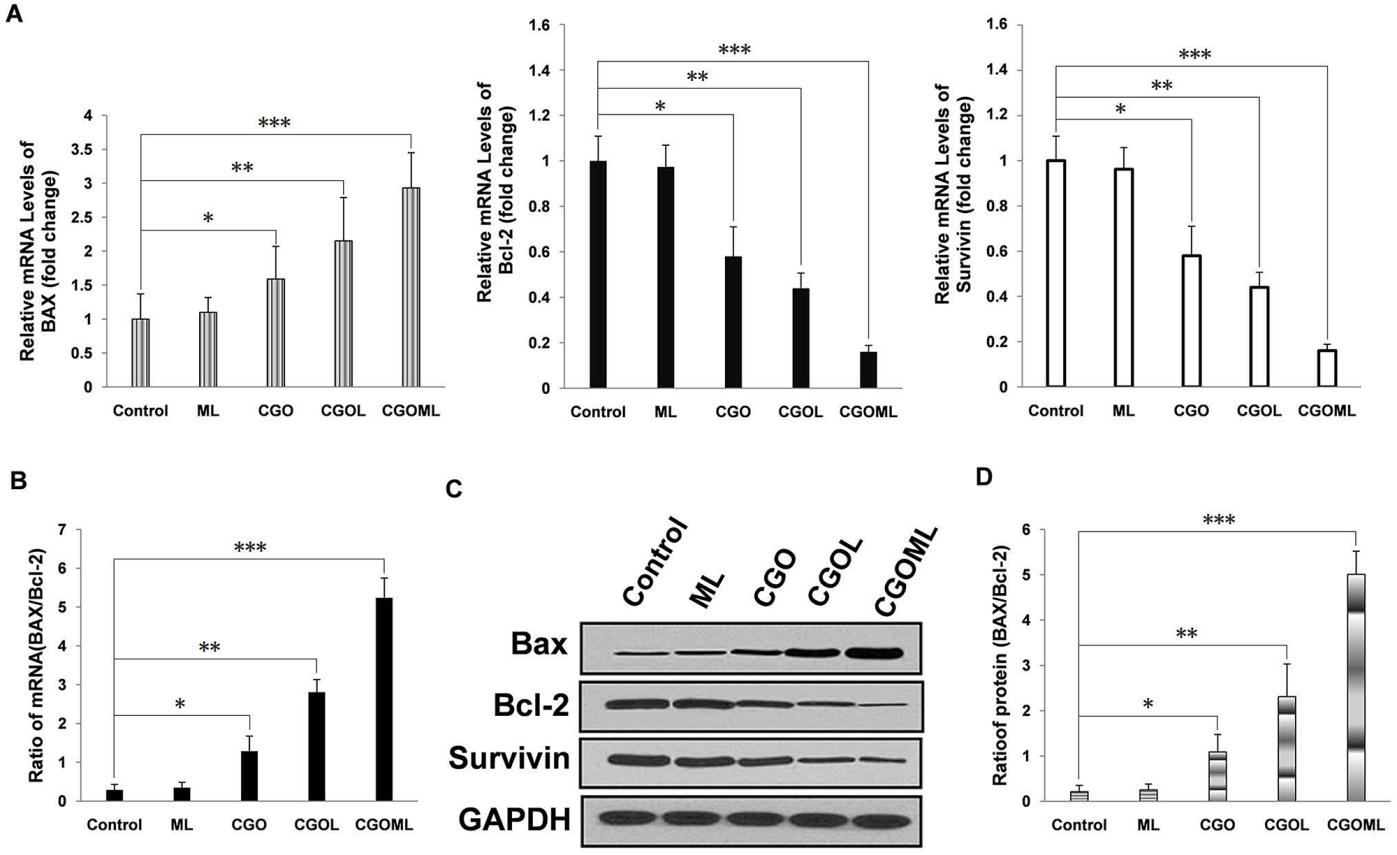
CGOML activate cell apoptosis in nude mice bearing breast cancer (MCF-7) Notes: **A.** RT-qPCR results show the mRNA levels of BAX, Bcl-2 and Survivin in tumors collected from mice of 5 different groups (Control, CGO, CGOL, ML, and CGOML groups). The left panel exhibits the relative mRNA levels of BAX in the 5 groups, the middle panel exhibits the relative mRNA levels of Bcl-2 in the 5 groups, whereas the right panel exhibits the relative mRNA levels of Survivin in the 5 groups. **B.** The mRNA level of BAX/Bcl-2 ratio was determined from results of (A) in each group. **C.** Western blot analysis shows BAX-, Bcl-2- and Survivin protein levels in tumor tissues collected from nude mice in different groups (Control, CGO, CGOL, ML, and CGOML). **D.** The protein levels of BAX/Bcl-2 ratio was calculated from results of (C). *p < 0.05, **p < 0.02, ***p < 0.01, significantly different from control group.

We first tested the transcription levels of Bcl-2, Survivin, and BAX in the tumor tissues. The CGOML group showed the highest BAX mRNA level among all groups (Figure 7A left panel). The CGO and CGOL groups also exhibited evidently higher BAX mRNA levels than the control and ML groups. No obvious difference was observed between the ML and control groups. Conversely, the CGOML group showed the lowest Bcl-2 mRNA level among all groups. The CGO and CGOL groups also exhibited obviously lower Bcl-2 mRNA levels than the ML and control groups (Figure 7A, middle panel). Similar to the case of Bcl-2, the CGOML group showed the lowest survivin mRNA level among all groups. The CGO and CGOL groups also had obviously lower Bcl-2 mRNA levels than the ML and control groups (Figure 7A, right panel). More importantly, the ratio of Bax to Bcl-2, but not their absolute amount, is an important predictive index of the apoptosis of breast cancer cells. Figure 7B shows that the mRNA level of BAX/Bcl-2 ratio in the CGOML group was approximately 18 times higher than that in the control group. The mRNA levels of BAX/Bcl-2 ratio in the CGO and CGOL groups were 4.45 and 9.69 times than that in the control group, respectively. Figure 7B also indicates no significant difference in the mRNA level of BAX/Bcl-2 ratio between the ML and control groups. Hence, these data suggested that apoptosis signaling pathways were also activated in mice bearing MCF-7 after drug treatment. The CGOML group exhibited the highest effects on tumor cell-apoptosis among all groups because of its high transport ability and targeting efficiency.

We subsequently measured the expression levels of BAX, Bcl-2, and survivin in all tumor tissues using Western blot. The results of Western blot were consistent with the results of mRNA level, which fully demonstrated that CGOML can markedly reduce the tumor size by inducing in vivo apoptosis of MCF-7 cells by downregulating Bcl-2, and survivin expression and upregulating BAX expression (Figures 7C and 7D).

## DISCUSSION

Breast cancer is the second most common cancer and remains a major cause of morbidity and mortality in women worldwide [28–29]. Aside from surgical excision, chemotherapy remains the backbone of current breast cancer treatment. However, its clinical use is limited by numerous drawbacks, which require novel therapies based on various combinations of anticancer drugs and procedures [30]. GemOx is a promising treatment strategy in breast cancer treatment because of its non-cross-resistance, synergistic antitumor activity, and tolerability of both drugs. However, GemOx still displays several side effects, such as neutropenia, anemia, thrombocytopenia, and neurosensitive toxicity [31].

Mikhail V Blagosklonny combined several drugs to achieve selectivity and efficacy of tumor therapy to improve tissue selectivity of drug [32–34]. However, this strategy cannot control the release of drug at the target area. Another strategy is to encapsulate the drug into a delivery system capable of guiding it to a target site [35]. Especially magnetoliposome, liposome-enveloped ferroferric oxide, possess the capability of target and sustained-release. Under the guidance of a magnetic field after intravenous administration, the drug-containing magnetoliposome can preferentially deliver the drug to tumors in vivo. Moreover, this drug delivery system can effectively control the release of drug at the target area. These properties enhance the stability of drugs, reduce drug dose, and alleviate drug toxicity [19, 26, 36–39].

In this study, we prepared GML or OML using reverse-phase evaporation combined with water bath ultrasonication. During preparation, the encapsulation of gemcitabine or oxaliplatin into the MLs prevented the digestion of gemcitabine or oxaliplatin in the blood or macrophages. We optimized the process conditions by a series of orthogonal experiments to achieve the ideal GML or OML. The results showed that Chol content was positively correlated with the mean diameters of GML and OML, making these particles more rigid [40]. DMPG, which is a negatively charged lipid, was necessary to make stable connections between ML and gemcitabine (or oxaliplatin). Figure 3 shows that our novel preparation process with an optimized lipid composition significantly increased the encapsulation efficiency and stability of GML (or OML).

Controlled release is one of prominent advantages of drug-containing magnetoliposome for treating cancer. GML and OML can weaken the effect of the sudden release of drug and remarkably increase the releasing time of the drug (Supplementary Figure S1). This finding may be ascribed to the strength of the drug-liposomal lipid interaction, fluidity of the bilayer, and half-life of the liposomal outer shell [41, 42].

Biomaterials, as carriers of drugs, should possess good stability and be safe to the blood, tissues, or immune system [43]. Thus, we also evaluated the stability and safety of GML and OML by hemolytic testing, acute toxicity testing, as well as pharmacokinetics and tissue distribution in vivo. The hemolytic and acute toxicity testing indicated that both GML and OML are safe. The pharmacokinetics and tissue distribution tests demonstrated that an external magnetic field prevented GML or OML from being absorbed by RES in the circulation, resulting in the accumulation of GML or OML in a targeted tissue (Figure 4).

**Figure 8:**
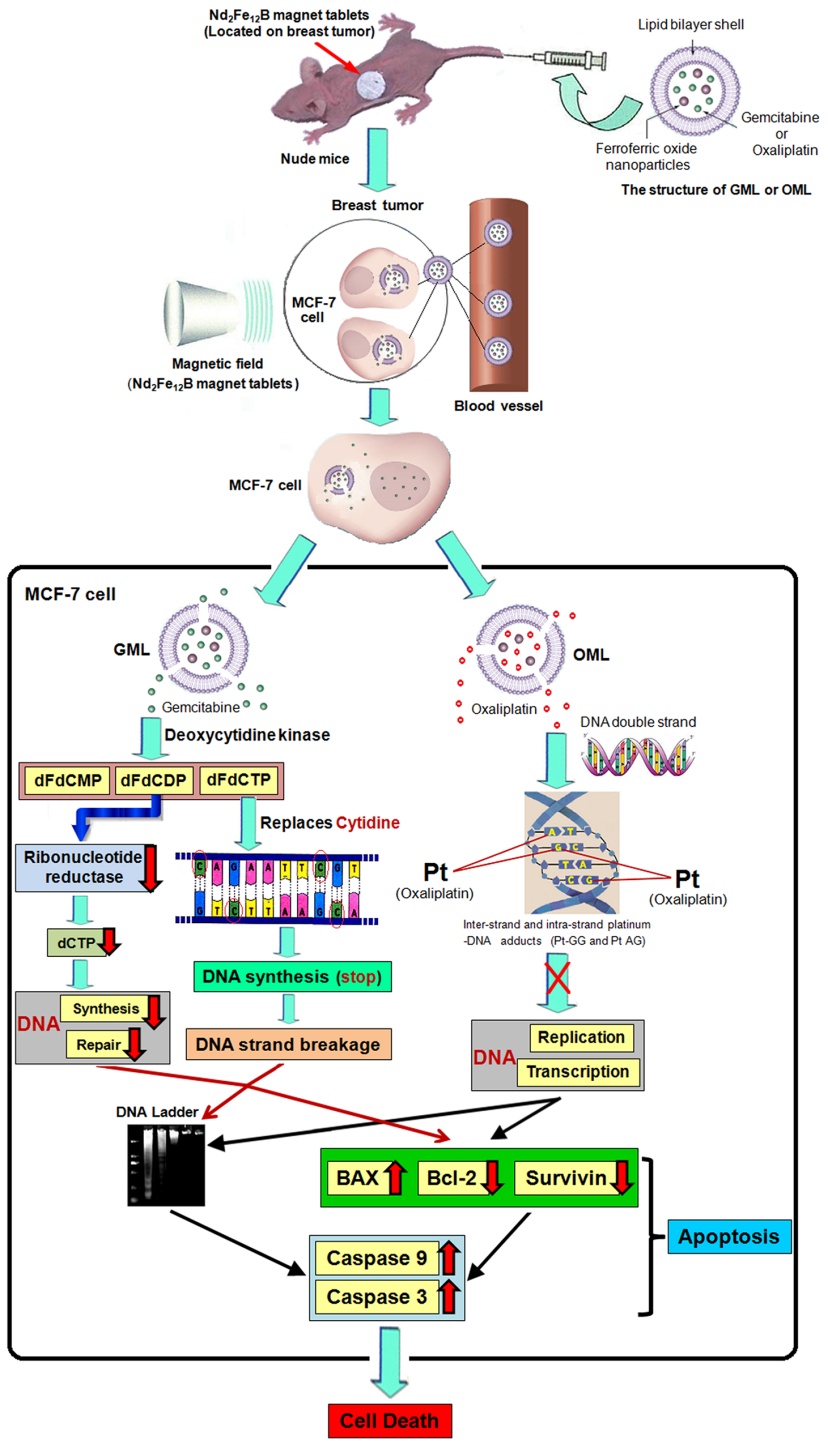
Proposed model of targeting therapy and signaling pathway involved in CGOML-induced apoptosis of MCF-7 cells: Nude mice bearing breast cancer (MCF-7) received intravenous injections of GML (35 μg of Gemcitabine/g) at day 1, 5 and then these mice also received intravenous injections of OML (5 μg of oxaliplatin/g) at day 3, 7; magnetic field (5000 GS) was applied to the tumor surface for 30 min after every injection The magnetic properties of GML or OML particles guide the gemcitabine or oxaliplatin to the tumor area of nude mice under an external magnetic field (Nd_2_Fe_12_B magnet tablets). GML or OML has excellent half-life periods and can be gradually biodegraded in MCF-7 cells, lead to gemcitabine of GML (or oxaliplatin of OML) slowly released in target cells. In MCF-7 cells, OML inhibited the replication and transcription of DNA by forming inter-strand and intra-strand platinum-DNA adducts (Pt-GG and Pt-AG), while GML prevented the synthesis and repair of DNA by inhibiting activation of ribonucleotide reductase, and stopped the the synthesis of DNA and broken strands of DNA by replacing the cytidine of DNA strands with dFdCTP. It leads to the phenomenon of DNA ladder, increase of BAX and decrease of Bcl-2 and Survivin, eventual activation of caspase-9 and caspase-3 cause cell apoptosis in MCF-7 cells, resulting in MCF-7 cell death.

Cell experiments demonstrated that CGOML significantly inhibited MCF-7 cell proliferation. The guidance of an external magnetic field facilitated the entry of CGOML into the tumor cell compared with CGOL, and the magnetic field partially retarded tumor growth. Moreover, animal studies indicated that CGOML under the guidance of an external magnetic field markedly reduced the tumor size of nude mice bearing human breast cancer (MCF-7) (Figures 5 and 6). Several drugs used for curing malignancies were reported to trigger apoptotic pathways [41]. Bax, a member of the Bcl-2 family, is an apoptosis promoter. By contrast, survivin and Bcl-2 are apoptosis inhibitors and can block programmed cell death [44, 45]. Thus, the Bax/Bcl-2 ratio can be used to evaluate the sensitivity of cells apoptosis [45, 46]. In this study, nude mice with MCF-7 was treated with CGOML under the guidance of an external magnetic field. Then, we assessed the relevance of Bax and Bcl-2 using RT-QPCR and Western blot analysis. Figure 7A and 7B show that, among the groups examined, the CGOML group exhibited the lowest mRNA expression of Bcl-2 and survivin and the highest mRNA expression of Bax. Western blot analysis indicated similar experimental results in the protein levels of the three genes (Bax, Bcl-2, and survivin) (Figure 7C and 7D). All results proved that gemcitabine and oxaliplatin were specifically delivered to the tumor site and triggered MCF-7 cell apoptosis, which inhibited tumor growth in nude mice with MCF-7. Thus, a possible mechanism of CGOML in the treatment for breast cancer is shown in Figure 8.

In conclusion, this study confirms that CGOML regimen exhibits the synergistic effect of gemcitabine and oxaliplatin in breast cancer treatment. Our study greatly improved the capability of target, sustained-release, and stability of drug in therapies. Therefore, the targeted therapy of CGOML for breast cancer can be potentially used for clinical treatment and requires further investigation.

## MATERIALS AND METHODS

### Cells and animals

MCF-7 cell line was purchased from the ATCC (Manassas, USA). The cells were cultured in RPMI 1640 medium (Invitrogen, USA) supplemented with 10% FBS (Sigma, USA), 100 U/mL penicillin, and 50 μg/mL streptomycin (Mediatech, USA) and incubated at 37°C with 5% CO_2_.

New Zealand rabbits (1.8–2.5 kg), ICR mice (18–22 g), and six-week-old athymic BALB/Ca nu/nu female mice (18–20 g) were purchased from the Shanghai Laboratory Animal Center of Chinese Academy of Sciences (Shanghai, China). BALB/Ca nu/nu micewere housed at constant room temperature under specific pathogen-free conditions and provided with a 12 h: 12 h light/dark cycle, standard rodent diet, and water ad libitum. All animal experiments were approved and evaluated by the Animal and Ethics Review Committee of Wenzhou Medical University (Wenzhou Medical University Policy and Welfare Committee, Document ID: WMU-2011-AP-0013).

### Compounds and reagents

PC, Chol, DMPG, chloroform, and Triton X-100 were supplied from Sigma-Aldrich (Saint Louis, USA). Sephadex-G-50 was acquired from Pharmacia Fine Chemicals (Uppsala, Sweden). Gemcitabine was purchased from Lilly (France), and oxaliplatin was purchased from Sigma (Saint Louis, USA). Fe_3_O_4_ (10 nm) particles weresupplied from Southwest Institute of Applied Magnetics of China (Chengdu, China). MTT cell proliferation assay kits were received from ATCC (Manassas, USA). Enhanced Apoptotic DNA Ladder Detection Kit was purchased from BioVision (Milpitas, CA, USA). A caspase-3 colorimetric assay kit was purchased from BioVision (USA). Trizol reagent, DNase I enzyme, SuperScript® III Reverse Transcriptase, andSYBR Green PCR Master Mix were purchased from Invitrogen (Carlsbad, CA, USA). BCA protein assay kits were acquired from Pierce (Rockford, USA). PVDF membranes were acquired from Bio-Rad Laboratories (Hercules, USA). Antibodies, namely, rabbit anti-human Bcl-2, BAX, survivin, GAPDH, and goat anti-rabbit IgG-HRP, were purchased from Santa Cruz Biotechnology (Santa Cruz, USA). ECL kits were supplied from Amersham Pharmacia Biotech (Piscataway, USA). All other reagents used were of analytical grade.

### Preparation, morphology, and biophysical characterization

GML was prepared by a combined method of reverse-phase evaporation and water bath ultrasonication, as described by Ye [41] but with slight modifications. In this study, the dry lipid film was hydrated using a solution of gemcitabine dissolved in PBS and 10 nm Fe_3_O_4_. GML was separated from Fe_3_O_4_ particles by Sephadex G-50 mini columns after magnetic sorting. Gemcitabine liposome (GL) was prepared using the same method but without ultrafine magnetite. ML was prepared following the same procedure but without drug addition (Figure 2). Similarly, the aforementioned method was applied to prepare and purify OML and oxaliplatin liposome (OL), and oxaliplatin was dissolved in 5% glucose (2 mg/mL) during preparation. Purified GML, OML, GL, OL, and ML were sterilized using 15 kGy of radiation with ^60^Co-γ rays and stored at 4°C. The particle sizes and encapsulation efficiencies were compared before and after irradiation.

In addition, the morphology, mean size, and size distribution of GML or OML were determined as described by Ye [41].

### Quantification and entrapment efficiency

Gemcitabine or oxaliplatin content was analyzed using HPLC. Gemcitabine content was analyzed using a symmetry C18 (5 μm; Hewlett-Packard) column equipped with a C18 guard column (5 μm, Hewlett-Packard) at 25°C with a mobile phase containing 40 mmol/L ammonium acetate buffer (pH 5.5)- acetonitrile (97.5:2.5, v/v) at a flow rate of 0.8 mL/min. The sample injection volume was 20 μL, and gemcitabine was detected using a UV detector at a wavelength of 273 nm. Oxaliplatin content was analyzed on a Hypersil BDS C18 column (250 mm × 4.6 mm × 5 μm, Thermo) using a mobile phase consisting of methanol–water (5:95, v/v) with UV detection at 250 nm and a flow rate of 1 mL/min. The column was maintained at 25°C throughout the analysis, and 20 μL of sample were injected. Different concentrations of free gemcitabine solution (2.0, 3.5, 5.0, 10.0, 25.0, 50.0, and 100.0 μg/mL) were used to calculate the standard curves. Gemcitabine content was determined and calculated. The precision of HPLC was evaluated using the relative standard deviation and the recovery rate. GML was separated from ferrofluid particles and free gemcitabine by using Sephadex G-50 mini-columns after magnetic sorting. Then, GML was directly dissolved in 10% Triton X-100 solution to destroy the liposome membrane and measure the amount of gemcitabine in the GML. Encapsulation efficiency (%) = C2/C1 × 100, where C1 is the total amount of gemcitabine in the solution before chromatography, and C2 is the amount of gemcitabine in GML. Similarly, oxaliplatin was quantified, and OML entrapment efficiency was determined using the aforementioned method. Different concentrations of free oxaliplatin solution (12.2, 24.4, 36.6, 48.8, 61.0, and 122.0 μg/mL) were used to calculate the standard curves.

### Stability of GML and OML formulations

Three batches of GML were prepared by reverse-phase evaporation, followed by ice-water bath ultrasonication. A sample (2 mL) was lyophilized with 5% mannitol as a protective agent and then stored at room temperature. Samples were then collected at 1, 2, and 3 months, and their encapsulated efficiencies were again determined by HPLC. Their morphologic and particle sizes were also characterized by TEM to analyze the stability of formulations. The same method was used to determine the stability of OML formulation.

### In vitro controlled-release of GML and OML

The release behavior of the drugs from GML and OML at 37°C was studied by the dialysis method. The dialysis tube (14,000 MWCO) containing 1 mL of OML was transferred to a beaker containing 50 mL of release medium (5% glucose solution) by maintaining a temperature at 37°C with continuousstirring at 100 rpm. Sink condition was maintained by periodicallyremoving 1 mL of the sample and replacing an equal volume of 5% glucose solution at 1, 1.5, 2, 2.5, 3, 4, 5, 6, and 8h. The amount of oxaliplatin released was analyzed with HPLC. A similar release study was performed using free oxaliplatin (as control group) in 5% glucose solution. The experiments were performed in triplicates.

The same method was used to examine the release of GML, but the MWCO ofthe dialysis tube was 10,000.

### Hemolytic test

Fresh rabbit blood was collected and prepared to 2% erythrocyte suspension to determine the biological safety of GML (1.5 mg gemcitabine/mL) to red blood cells. Then, eight tubes were arranged as follows:

tube 1: 5 mL of 2% erythrocyte suspension (A) + 0.1 mL of GML+4.9 mL of saline;
tube 2: 5 mL of A+0.2 mL of GML + 4.8 mL of saline;
tube 3: 5 mL of A+0.3 mL of GML + 4.7 mL of saline;
tube 4: 5 mL of A+0.4 mL of GML + 4.6 mL of saline;
tube 5: 5 mL of A+0.5 mL of GML + 4.5 mL of saline;
tube 6: 5 mL of A+0.6 mL of GML + 4.4 mL of saline;
tube 7: 5 mL of A+5 mL of saline (as a negative control);
tube 8: 5 mL of A+5 mL ofdouble-distilled water (as a positive control).

All tubes were mixed and incubated in a water bath at 37°C for 1 h and centrifuged for 5 min at 2,500 rpm. Then, the supernate was measured at 545 nm by Shimadzu UV2550 UV-vis spectrophotometer (Kyoto, Japan). Hemolysis rate was calculated as follows: hemolysis rate (%) = (ODt−ODnc)/(ODpc−ODnc) × 100, where ODt, ODnc, and ODpc are the absorbance values of the test group, negative control, and positive control, respectively. Similarly, the biological safety of OML (0.25 mg oxaliplatin/mL) to cells was determined using the aforementioned method.

The effects of GML and OML on platelets and leukocytes were examined using the method as described by Ye [41].

### Acute toxicity test

A total of 40 clean ICR mice (6–8 weeks old) were randomly divided into four groups (n=10). The control group received intravenous injections of saline, whereas the low-dose, middle-dose, and high-dose groups received intravenous injections of 9, 18, and 35μg gemcitabine/g, respectively. The feeding conditions were as follows: room temperature, 60% humidity, natural light for 12 h each day, standard solid composite feed stuff, and tap water ad libitum. The daily growth behavior of all mice the following week was observed and recorded. The hearts, breasts, spleens, lungs, kidneys, stomachs, intestines, and thymi were collected after the mice were sacrificed by cervical dislocation for pathologic anatomical examination. In the same way, the biological safety of OML (containing oxaliplatin at 1.25, 2.5, and 5 mg/kg) to animals was determined using the aforementioned method. The control group of the OML group was intravenously injected with 5% glucose solution.

### Pharmacokinetics and tissue distribution

A total of 30 ICR mice (18–22 g) were randomly divided into three groups. Group 1 was injected with free gemcitabine or oxaliplatin. Group 2 [GML (+) or OML (+)] was injected with GML or OML. The heads of the mice were under continuous external magnetic field (Nd_2_Fe_12_B permanent magnet tablets) of 5000 GS for 30 min. Group 3 [GML (−) or OML (−)] was injected with GML or OML, and the heads of mice were not treated with an external magnetic field. Approximately 35 mg gemcitabine/kg body weight or 5 mg oxaliplatin/kg body weight were administered via tail vein injection to all experimental mice in all groups. Blood samples (100 μL) were collected from the retro-orbital plexus at various times (gemcitabine: 0.1, 0.25, 0.5, 1, 2, 4, 8, 12, 24, 36, 48, and 60 h; oxaliplatin: 0.25, 0.5, 1, 2, 6, 12, 24, and 48 h). The plasma samples were collected after centrifugation at 3,000 rpm for 10 min and then stored immediately at −20°C. In the tissue-distribution study, 30 additional ICR mice were randomly divided into three groups and then treated as previously described. Approximately 90 min after injection of gemcitabine or oxaliplatin, the kidneys, liver, spleen, heart, lungs, and brain of each mouse was rapidly excised following sacrifice and immediately washed twice with normal saline (or 5% glucose solution). The samples were wiped with a filter paper, weighed, and homogenized with 1.0 mL of normal saline (or 5% glucose solution). The sample was centrifuged for approximately 10 min at 3,000 rpm. The supernatant was collected and then stored immediately at −20°C. The plasma and tissue samples were prepared and analyzed as described earlier. A volume of 20 μL of samples was directly injected into the HPLC system for analysis.

### Cellular uptake and detection of platinum-DNA adduct

The cellular uptake of oxaliplatin and platinum-DNA adduct was measured as described previously [47] but with slight modifications. MCF-7 cells were divided into three groups with 5× 10^6^ MCF-7 tumor cells per group and treated as follows: control group, 5% glucose solution at 37°C for 6 h; oxaliplatin group, 20 μg oxaliplatin/mL at 37°C for 6 h; OML group, 20 μg oxaliplatin/mL at 37°C for 6 h. Then, 1.6 mL of resuspension liquid of cell pellet with PBS in each group was divided into three parts and tested for a given parameter, as follows: 0.3 mL, cellular uptake; 0.2 mL, protein concentration; and 1.1 mL, DNA concentration and DNA adduct.

### Cytotoxicity assay

The in vitro cytotoxicities of GML and OML were assessed by MTT assay, as described previously [41]. The MCF-7 cells (1×10^4^ per well) were plated in a 96-well plate in RPMI-1640 medium. All wells were divided into control (RPMI 1640 medium), ML, CGO (gemcitabine), CGOL (GL), and CGOML (GML) groups. The wells containing gemcitabine, GL, and GML were further divided into six subgroups and incubated with different concentrations of gemcitabine (0.5, 1, 2, 4, 8, 16, and 32 μg/mL). A magnetic field (Nd_2_Fe_12_B magnet tablets) of 5000 GS was applied to the bottom of the plate for 30 min after the drugs were added. The cells were cultured for 12 h, and then oxaliplatin, OL, and OML were also added into these wells to finally form the CGO, CGOL, and CGOML groups, respectively. The wells containing oxaliplatin, OL, and OML were further divided into six subgroups and incubated with different concentrations of oxaliplatin (0.5, 1, 1.5, 2.5, 5, 10, and 20 μg/mL). Then, a magnetic field of 5000 GS was applied to the bottom of the plate for 30 min. MTT reagent (10 μL) was added to each well after the cells were further cultured for 36 h. The plate was again incubated for 4 h. DMSO (150 μL) was then added to each well to dissolve the formazan crystals. The absorbance of each well was read at 570 nm on a microplate reader.

### Caspase-3 activity test

MCF-7 cells were divided into five groups, and then, caspase-3 colorimetric assay kit was used to detect caspase-3 activity in MCF-7 cells as described previously [41].

Each group received treatment, as follows:

I Control group received RPMI 1640 medium. Then, a magnetic field (Nd_2_Fe_12_B magnet tablets) of 5000 GS was applied to the bottom of the plate for 30 min.
II ML group received ML (0 μg of gemcitabine/mL, 0 μg of oxaliplatin/mL). Then, a magnetic field of 5000 GS was applied to the bottom of the plate for 30 min.
III CGO group received 32 μg of gemcitabine/mL. Then, a magnetic field of 5000 GS was applied to the bottom of the plate for 30 min. Then, 20 μg of oxaliplatin/mL was administered after 12 h. A magnetic field of 5000 GS was again applied to the bottom of the plate for 30 min.
IV CGOL group received GL (32 μg of gemcitabine/mL), and then a magnetic field of 5000 GS was applied to the bottom of the plate for 30 min. Then, OL (20 μg of oxaliplatin/mL) was administered after 12 h. A magnetic field of 5000 GS was again applied to the bottom of the plate for 30 min.
V CGOML group received GML (32 μg of gemcitabine/mL), and a magnetic field of 5000 GS was applied to the bottom of the plate for 30 min. Then, OML (20 μg of oxaliplatin/mL) was also administered after 12 h. A magnetic field of 5000 GS was again applied to the bottom of the plate for 30 min.

### DNA fragmentation analysis

MCF-7 cells were divided into five groups (control, ML, CGO, CGOL, and CGOML group), with each group treated as described earlier. An enhanced apoptotic DNA ladder detection kit was used to detect apoptosis induction of MCF-7 cells in accordance with the manufacturer's recommended protocol. The cells were further incubated at 37°C for 24 h. Adherent and floating cells were recovered. DNA was isolated and evaluated for fragmentation as described previously [48]. DNA samples were separated using 1.5% agarose gel (containing 1% GoldView™) electrophoresis. Finally, UV gel documentation system (Bio-Rad, Hercules, CA, USA) was applied to examine and photograph the gel.

### Targeting therapy on nude mice bearing breast cancer

MCF-7 cells (1 × 10^7^ cells) were inoculated subcutaneously into the right flank of female BALB/c nude mice to develop tumor-bearing mice. Then, 30 nude mice bearing MCF-7 with tumors 6–8 mm in diameter were randomly and equally grouped. The start of the day treatment was defined as day1. Two perpendicular diameters of tumors were measured on days 1, 2, 3, 5, 7, and 10. Finally, the mice were killed, and their tumors were collected on day 10. Tumor volume and tumor growth inhibition rate were calculated using the method described by Ye [41].

Each group received treatment, as follows:

I Control group (n=6) received intravenous injections of normal saline (or 5% glucose solution) on days 1, 3, 5, and 7. Then, a magnetic field (Nd_2_Fe_12_B magnet tablets, 5000 GS) was applied to the tumor surface for 30 min after every injection.
II ML group (n=6) received intravenous injections of ML [0 μg of gemcitabine/g (body weight, the same as below) and 0 μg of oxaliplatin/g] on days 1, 3, 5, and 7. Then, a magnetic field (5000 GS) was applied to the tumor surface for 30 min after every injection.
III CGO group (n=6) received intravenous injections of 35 μg gemcitabine/g on days 1 and 5. Then, 5 μg oxaliplatin/g were also intravenously injected on days 3 and 7. Magnetic field (5000 GS) was applied to the tumor surface for 30 minafter every injection.
IV CGOL group (n=6) received intravenous injections of GL (35 μg of gemcitabine/g) on days 1 and 5. Then, OL (5 μg of oxaliplatin/g) were intravenously injected on days 3 and 7. Magnetic field (5000 GS) was applied to the tumor surface for 30 min after every injection.
V CGOML group (n=6) received intravenous injections of GML (35 μg of gemcitabine/g) on days 1 and 5. Then, OML (5 μg of oxaliplatin/g) were intravenously injected on days 3 and 7. Magnetic field (5000 GS) was applied to the tumor surface for 30 min after every injection.

### Real-time quantitative RT-PCR

Mice were sacrificed by cervical dislocation on day 10 after treatment as described earlier. Breast tumors were collected and immediately placed in liquid nitrogen for further experiments. Total mRNA was extracted from tumor tissues using Trizol reagent, and then SuperScript® III Reverse Transcriptase was used to obtained cDNA of mRNA. Real-time quantitative PCR reactions were performed with SYBR Green PCR Master Mix in accordance with the manufacturer's instructions. The sequences of the primers were as follows: Bcl-2, sense, 5′-TTGGATCAGGGAGTTGGAAG-3′, antisense, 5′-TGT CCCTACCAACCAGAAGG-3′; Survivin, sense, 5′-CAT GGCTACCAGCACCTGAAAG-3′, antisense, 5′-TTTGGCT TGCTGGTCTCTTCTG-3′; Bax, sense, 5′-AGGATGC GTCCACCAAGAAG-3′, antisense, 5′-GAGTCTCACCC AACCACCCT-3′; and GAPDH (as an internal standard), sense, 5′-GCCAAAAGGGTCATCATCTC-3′, antisense, 5′-GCCTTCAACGCCTGCTTC-3′. Amplification mixes (50 μL) contained the sample DNA (reverse-transcribed cDNA) or ddH_2_O as a negative control (template-free controls), primers, ddH_2_O, and 25 μL of SYBR Green PCR Master Mix. PCR amplification was conducted as follows: for Survivin, an initial denaturation of 95°C for 5 min, 30 cycles (30 s at 94°C, 30 s at 55°C, and 45 s at 72°C), and 10min at 72°C for a final elongation step; for Bcl-2, BAX, and GAPDH, an initial denaturation of 95°C for 5 min, 30 cycles (30 s at 95°C, 30 s at 55°C, and 60 s at 68°C), and 10min at 68°C for a final elongation step. The relative amount of Bcl-2 (or Survivin or BAX) mRNA was normalized to an internal control GAPDH.

### Western blot analysis

Mice were sacrificed by cervical dislocation on day 10 after treatment as described earlier. Breast tumors were harvested, cut into small pieces on ice, and placed into lysis buffer containing solution A. The samples were then vortexed, homogenized, and centrifuged. Protein concentrations in the supernatant were determined using BCA protein assays kit (Pierce, Chemical Co., USA). Each protein sample (approximately 50 μg) was electrophoresed and then further transferred onto polyvinylidene difluoride membrane. The bots were blocked with fresh 5% nonfat milkin TBS buffer at 4°C overnight and incubated with rabbit anti-human Bcl-2 (or Survivin or BAX) polyclonal antibody as the primary antibody at 1:500 ratio for 2 h. After three washes with TBST, the blots were incubated with horseradish-peroxidase conjugated goat anti-rabbit IgG as the secondary antibody for 1.5 h. The transferred proteins were incubated with enhanced chemiluminescence buffer followed by visualization with X-ray film. The density of the immunoreactive bands was analyzed using NIH Image version 1.61 (National Institutes of Health, Bethesda, USA). The quantities of Bcl-2 (or Survivin or BAX) protein were standardized against GAPDH as an internal control.

### Statistical analysis

One-way ANOVA and 2 sample t-tests were employed to analyze significant differences between sets of data. P values less than 0.05 were considered statistically significant. Statistics were performed using the standard statistical software SPSS 22.0.

## SUPPLEMENTARY FIGURES AND TABLES


