# Supplementary material for: Combination of gemcitabine-containing magnetoliposome and oxaliplatin-containing magnetoliposome in breast cancer treatment: A possible mechanism with potential for clinical application
Source: Oncotarget. 2016 May 27;7(28):43762–78. doi: 10.18632/oncotarget.9671 (PMC5190058; doi:10.18632/oncotarget.9671)
Supplement: Supplementary file 1 [file oncotarget-07-43762-s001.pdf]

Combination of gemcitabine-containing magnetoliposome and oxaliplatin-containing magnetoliposome in breast cancer treatment: A possible mechanism with potential for clinical application

SUPPLEMENTARY FIGURES AND TABLES

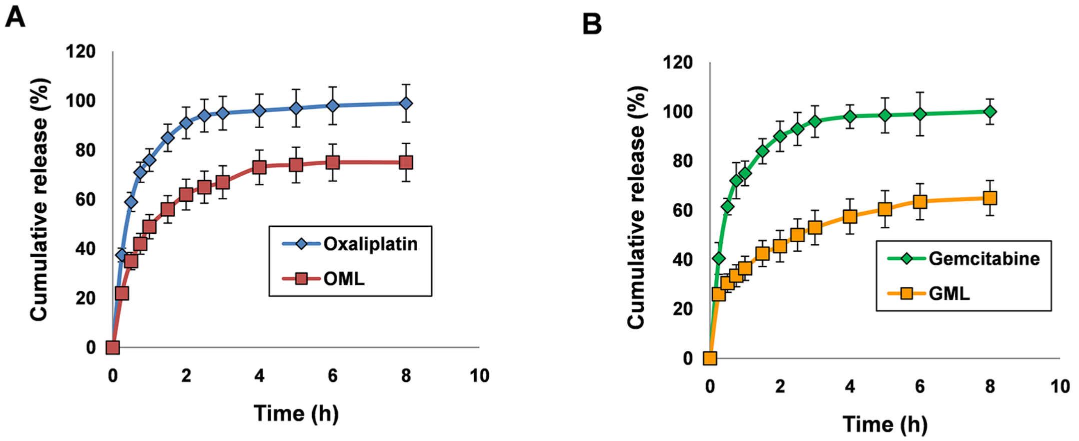

Supplementary Figure S1: Analysis of controlled drug release of GML and OML. A. In vitro release profile of Oxaliplatin and OML at 37°Cin 5% glucose solution by the dialysis method. Each point represents the mean±SD and p≤0.05 was considered to be statistically significant; **B.** In vitro release profile of Gemcitabine and GML at 37°Cin 5% glucose solution by the dialysis method. Each point represents the mean±SD and p≤0.05 was considered t o be statistically significant.

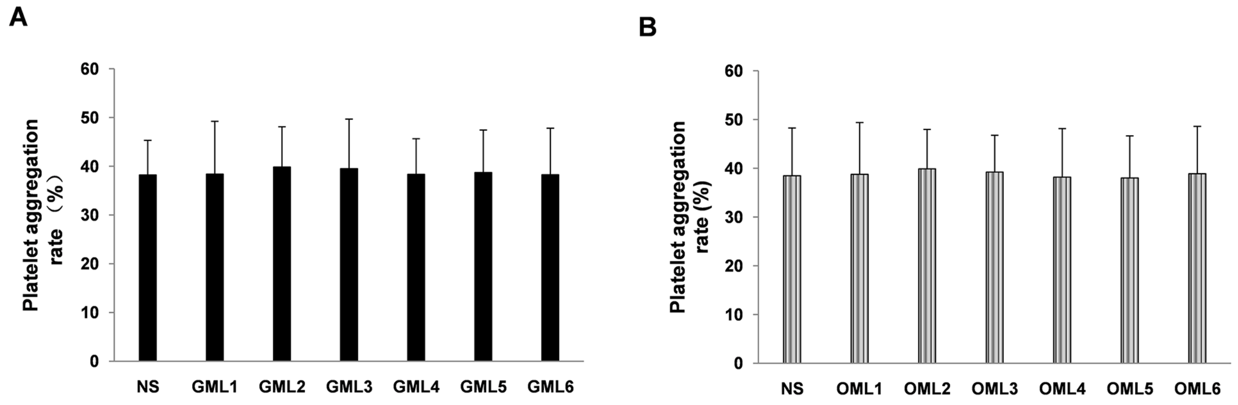

Supplementary Figure S2: Effects of GML, OML on platelets. Notes: A. Platelet-aggregation rates from seven different groups were shown. Here, group 1 is the control group (NS), in which platelets were treated with saline, whereas groups 2-7 are the platelets treated with GMLcontaining the following concentrations of gemicitabine: 0, 0.3, 0.6, 0.9, 1.2, 1.5, 1.8 mg/mL. **B.** Platelet-aggregation rates from seven different groups were shown. Here, group 1 is the control group (NS), in which platelets were treated with saline, whereas groups 2-7 are the platelets treated with OML containing the following concentrations of oxaliplatin: 0, 0.05, 0.10, 0.15, 0.20, 0.25, 0.30 mg/mL.

SupplementaryTable S1: Effects of GML on numbers and functions of leukocytes in peripheral blood (n=10, $\bar{x}$ ±s)

Group	gemicitabine (mg/mL)	WBC/$10^9 \cdot L^{-1}$ (%)	PMNS/$10^9 \cdot L^{-1}$ (%)	LYM/$10^9 \cdot L^{-1}$ (%)	Percentage of NBT positive cells/%
NS	0	15.29±3.02	3.11±0.98	11.81±2.66	31.30±8.90
GML1	0.3	15.05±3.01	3.09±1.12	11.51±3.12	31.23±9.00
GML2	0.6	15.44±3.00	2.96±0.93	11.68±2.59	30.90± 7.30
GML3	0.9	15.98±2.52	3.22±1.15	11.97±2.90	31.70± 9.14
GML4	1.2	15.46±3.20	2.99±1.10	11.84±2.75	31.02± 7.16
GML5	1.5	15.78±2.87	3.00±1.03	11.71±2.83	30.80± 8.50
GML6	1.8	15.82±2.94	3.10±0.88	11.68±2.33	31.11± 7.87

Supplementary Table S2: Effects of OML on numbers and functions of leukocytes in peripheral blood (n=10, $\bar{x}$ ±s)

Group1	oxaliplatin (mg/mL)	WBC/$10^9 \cdot L^{-1}$ (%)	PMNS/$10^9 \cdot L^{-1}$ (%)	LYM/$10^9 \cdot L^{-1}$ (%)	Percentage of NBT positive cells/%
NS	0	15.20±2.99	3.21±1.06	11.12±3.30	31.22±7.82
OML1	0.05	15.29±3.24	3.14±1.17	11.30±3.22	30.85± 8.71
OML2	0.10	15.32±3.17	3.35±1.03	11.31±3.28	31.21±8.12
OML3	0.15	15.60±3.28	3.14±1.10	11.24±3.14	30.50± 7.63
OML4	0.20	15.71±3.56	3.23±1.20	11.61±3.42	31.48± 8.70
OML5	0.25	15.58±3.34	3.30±1.14	11.53±3.11	31.38± 7.54
OML6	0.30	15.64±2.90	3.21±1.31	11.65±3.27	31.73± 8.74
